# The tree of genomes: An empirical comparison of genome-phylogeny reconstruction methods

**DOI:** 10.1186/1471-2148-8-312

**Published:** 2008-11-12

**Authors:** Angela McCann, James A Cotton, James O McInerney

**Affiliations:** 1 Bioinformatics laboratory, Department of Biology, National University of Ireland Maynooth, Maynooth, Co. Kildare, Ireland

## Abstract

**Background:**

In the past decade or more, the emphasis for reconstructing species phylogenies has moved from the analysis of a single gene to the analysis of multiple genes and even completed genomes. The simplest method of scaling up is to use familiar analysis methods on a larger scale and this is the most popular approach. However, duplications and losses of genes along with horizontal gene transfer (HGT) can lead to a situation where there is only an indirect relationship between gene and genome phylogenies. In this study we examine five widely-used approaches and their variants to see if indeed they are more-or-less saying the same thing. In particular, we focus on Conditioned Reconstruction as it is a method that is designed to work well even if HGT is present.

**Results:**

We confirm a previous suggestion that this method has a systematic bias. We show that no two methods produce the same results and most current methods of inferring genome phylogenies produce results that are significantly different to other methods.

**Conclusion:**

We conclude that genome phylogenies need to be interpreted differently, depending on the method used to construct them.

## Background

Hundreds of genome sequencing projects have been completed [1], providing us with an abundant source of data to reconstruct phylogenetic relationships, but also with some novel problems in interpreting these data. The evolutionary history of any genome includes elements of gene duplication, gene loss, lineage sorting and horizontal transfer of genes, all of which have the ability to confound phylogeny reconstruction [2-4]. Against this background, a variety of genome-phylogeny methods have been developed. These vary in their approach, the input data they require and the interpretation of the result. However, to date, no study has been carried out that asks whether these methods are picking out fundamentally different signals or if they are more-or-less finding the same tree.

Current genome-level phylogeny methods can be split into two categories – sequence-based methods and gene-content methods. Analyses of sequence evolution predates gene-content methods simply because data for individual genes were available before data for completed genomes. Ubiquitously distributed ribosomal RNA (rRNA) genes have usually been used as surrogates for larger samples of individual genomes. These particular genes are popular for phylogenetic studies due to their plentitude, universally conserved structure and apparent resistance to horizontal gene transfer (HGT) [5]. In contrast, some methods are designed to include information from the evolutionary history of several individual genes. The supertree approach, for instance, involves the creation of individual trees from gene families and the amalgamation of these into one final supertree. Another sequence-based approach has involved the concatenation of alignments of several genes [6-8] usually with an effort being made to remove sequences that have an obvious history of HGT. Data concatenation should have the effect of minimizing stochastic effects due to small sample size and amplifying low signals, though gene concatenation is not without its problems [8,9].

The second group of methods uses variation in gene-content as the basis for phylogeny reconstruction. These approaches range from the use of similarity of gene-content [10-12] to the inclusion of the analysis of gene order data [13]. Usually a pairwise analysis of genomes in a set is carried out with a metric being computed that reflects the similarity between the genomes, this can be done using a maximum parsimony score, a threshold parsimony score (e.g. [14]) or deriving a phylogenetic distance. Finally phylogenetic hypotheses are generated based upon these scores.

If the process of evolution is indeed hierarchical or tree-like, then with increased sampling, all reasonable or consistent methods should converge on the same tree. Recent work has found a great deal of congruence between phylogenetic trees for different gene families in closely related organisms but a lack of congruence between gene trees from distant relatives [15,16]. This suggests that the pattern of inheritance of genes may indeed be largely vertical, or at least tree-like for parts of the reconstructable tree, but that this pattern is difficult to identify for deep-level relationships [17]. In other words, parts of life's history may not be reconstructable because of incorrect identification of orthologs, hidden paralogy, horizontal gene transfer events or the inability of methods based upon current evolutionary models to correctly reconstruct deep-level phylogenetic relationships. Ideally, a formal probabilistic model describing all of the many processes involved would allow us to both study these processes quantitatively and reconstruct phylogenetic relationships [18], but no such unifying models exist, and any such model would be complex and difficult to fit.

Given that a number of heuristic methods now exist for the inference of phylogenetic histories from genomic data, it is reasonable to ask whether these methods are likely to give fundamentally different answers. In this report, we have examined the similarity of the results we obtain when we use a variety of different organismal phylogeny reconstruction approaches on a real dataset. In reality, given the enormous number of genome-phylogeny methods, we have not tried to exhaustively explore all available methods. Instead, we have chosen exemplar methods that use different kinds of data. If all genome-phylogeny methods tend to return the same answers, then it probably does not matter which one is used; however, if on the other hand, different methods give different results, the choice of method becomes important.

We have used exploratory statistics in order to examine the phylogeny of *22 *diverse Archaea for which completed genome sequences are available. In particular, we wished to explore variation in the phylogenetic hypotheses from this dataset. Our comparisons involved using five distinct phylogeny reconstruction methods and their variants, giving a total of nine methods. Seven of these methods use large portions of each genome: four variants of the Conditioned Reconstruction (CR) method [11,19], two variants of the SHOT method [20] and a Supertree approach [21]. We have also examined reconstructions based on the 16S rRNA molecule and from a concatenated alignment of 31 genes involved in translation (the same genes used by [7]).

Particular attention is paid to CR in this report [11,19]. This is a method that has not been used extensively but which forms the basis of the "Ring of Life" hypothesis [11,19]. CR is based on the analysis of shared gene-content, with closely related species sharing a large proportion of genes whilst more distantly related species have fewer genes in common. The method is based on the formation of a matrix consisting of the four possible patterns of joint presence (P) or absence (A) of genes between any two genomes (PP, PA, AP, AA). The proportions of the first three are readily determined, but the shared absences will pose a problem for any Markov method using presence-absence data to infer phylogenetic signal. This is due to the fact that the analysis will only be carried out over all genes present in either genome one or genome two. The authors' solution is to introduce a conditioning genome; *i.e. *an additional genome that is used solely for reference purposes. Genes that are present in the conditioning genome, but absent in the two genomes under consideration provide an estimate of shared absence.

Along with their claim about insensitivity to HGT the authors also claim that the phylogenetic outcome of CR is not affected by the choice of conditioning genome [11,19]. However, there has been little work testing either of these conjectures, or examining the performance of CR in comparison to other methods [22]. An analysis was however preformed to determine if CR could differentiate between genome fusions or HGT events involved in the formation of mixed genomes [22], as the authors claimed it could. Bailey et al (2006) concluded that this was not possible and that CR can actually induce bias in ortholog sampling; they also showed using simulation studies that different conditioning genomes can result in different trees being derived for the same dataset. Another study of CR carried out by Spencer et al (2007), also showed that altering the conditioning genome in an analysis can affect bootstrap support for different tree topologies.

A problem that we encounter in this study concerns the issue of using different analysis methods and using different kinds of data. When we consider a single alignment, then it is usual to perform an initial analysis to find which model best fits the data [23,24]. Then using this model, the best phylogenetic tree or set of trees is found using some optimization procedure [25]. Therefore, the two variables will be the choice of alignment – whether to use a single gene [26,27] or concatenate several genes together [6-8] – and the choice of model. When using gene-content data, the choices will include the method of encoding gene-content and the way in which the encoded data is analysed [10-12]. For an analysis that includes phylogenetic supertree construction, the choices will center on the model that is used to construct phylogenetic trees from the alignments of orthologs and the method of inferring the supertree [28]. Clearly, it is difficult to carry out a study that uses all combinations of methods and also it is difficult to use an approach that holds all variables constant while only changing one at a time. In this study, we have chosen to use a representative sample of approaches and our analysis involved comparing the final sets of phylogenetic hypotheses. The overall objective of this work is not to identify which method yields the correct phylogeny, rather it is to ask whether the different methods are, in general, producing the same phylogenies.

## Methods

### Ortholog identification

A total of 22 fully sequenced Archaeal genomes were downloaded from the Cogent database [1]. A previously described greedy algorithm was used to identify orthologous families in these genomes [16]. Briefly, a random query sequence was chosen from the original database of 22 Archaeal genomes and homologous genes were identified as BLASTP hits [29] with an E-value of 10^-8 ^or less. The initial query sequence and all hits were then removed from the original Archaeal database and the process continued iteratively with a new query until all genes were assigned to a gene family. A total of 14,673 gene families were identified. From this initial set, 1,655 paralogous families were eliminated by removing all gene families with more than one representative from any genome. 11,864 phylogenetically uninformative gene families (fewer than four sequences) were also eliminated. Amino-acid sequences for each gene family were then aligned using ClustalW v1.83 [30] with all settings at their default values. Gblocks [31] was then used to remove highly variable positions from the alignment, these are potentially fast evolving or poorly aligned regions. In Gblocks [31], the maximum number of contiguous non-conserved positions allowed was set to 15 and the minimum length of a block was set to 8 amino acid positions. Following Gblocks site removal, those alignments that now had fewer than 150 amino acid positions remaining were excluded from further analysis. All remaining alignments were screened for the presence of phylogenetic signal using the Permutation Tail Probability (PTP) test [32,33]. Only 594 alignments passed the test (p < 0.01) and were retained. The presence or absence of these remaining single-copy gene families were scored in a matrix and provided the input for the gene-content based phylogenetic methods.

### Supertree

The 594 remaining alignments were analysed using Multiphyl [23]. This software reconstructs maximum-likelihood (ML) phylogenies for each gene family using the best-fitting empirical homogeneous model of amino-acid substitution, according to the Akaike Information Criterion (AIC). Multiphyl [23] distributes the model selection and tree search calculations across a network of computers. Tree space was searched using Nearest Neighbor Interchange (NNI) branch swapping and local branch length optimization, until convergence. Following gene tree estimation, a supertree was inferred from these gene trees using CLANN [21] with the Most Similar Supertree Algorithm (MSSA/DFIT) criterion [15], using the default heuristic search options. Non-parametric bootstrapping was carried out by sampling-with-replacement 100 pseudoreplicates of individual gene trees, using the default settings in CLANN [21], generating a supertree for each of these replicates and summarizing the results using a majority-rule consensus method.

### Conditioned Reconstruction

A matrix of presence and absence of gene families (see ortholog identification above) was analysed using a Java implementation of the CR algorithm [11,19], (software available on request). Our program implements a number of variants of the standard CR method. The first approach uses only a single conditioning genome, as originally proposed by the authors [11,19]. The conditioning genome is specified by the user and a phylogeny is inferred using paralinear/logdet distances [34,35]. The first variant of CR is called averaged (Avg) CR and does not require the *a priori *identification of a conditioning genome. In this case, every genome is used as the conditioning genome. In other words, when working out the distance between two genomes, every other genome acts the conditioning genome. The logdet distances derived using each conditioning genome are summed and the mean of this value gives the final distance between the two genomes of interest, this process is repeated for all pairs of genomes in the analyses. The second variant is an unconditioned distance approach (see [36]), this involves including a pseudo-conditioning genome in which every gene family is present (i.e. comprised entirely of the present state).

The final variant of CR analysed in this report, employed software created by Spencer et al., (2007). This program is based on a modified BIONJ algorithm [37], adapted to produce a supertree. The input to this program is a series of distance matrices, each derived using a different conditioning genome; in our case 22 different matrices were used, therefore all genomes in this analysis acted as the conditioning genome at one point. The modified BIONJ algorithm operates by firstly choosing a pair of taxa from each distance matrix that minimizes some criterion (see [36]). The best such pair across all the distance matrices are then selected and the subtrees containing these taxa are aggregated in all distance matrices; finally the distance matrices are updated. This process is continued iteratively until every taxon has been aggregated in every matrix and a supertree is produced. Spencer et al., (2007) provide two different approaches of the algorithm, both of which were used in this report. The first method is a vote-counting method that does not take into account differences in reliability between conditioning genomes. The second is an inverse-variance weighting scheme that does take into account differences in reliability between conditioning genomes.

In total four variants of CR are implemented in this study. With the exception of the modified BIONJ approach described above, all distance matrices were converted into phylogenetic trees using the neighbor-joining algorithm implemented in PHYLIP [38]. In addition, we constructed 100 bootstrap pseudoreplicates by resampling the original presence and absence matrix (see ortholog identification above).

### SHOT method

Two distance matrices were derived from the matrix of all orthologs (see ortholog identification) based on two variants of the SHOT method [20], by applying the formulae below:

(1) $$d_{1}=-\log(n_{pp}\frac{\sqrt{a^{2}+b^{2}}}{ab\sqrt{2}})$$

(2) $$d_{2}=-\log(\frac{n_{pp}}{\min(a,b)})$$

*n*_*pp *_is the number of gene families in common between the two genomes of interest and *a *and *b *are the number of gene families in each of the two genomes individually. Following derivation of a distance matrix, phylogenetic hypotheses were derived using the neighbor-joining method as described above. Bootstrap resampling was employed in order to examine variation in estimates from these approaches.

### Concatenated alignment

A concatenated alignment was built using the 31 genes used by Cicarelli et al., (2006; see table S2). These genes are largely involved in translation and have been described as having "indisputable orthology" in 191 species. The complete data matrix was obtained from the iTOL website [39] and all non-archaeal species were removed. Four genomes used in our study were absent from the Ciccarelli et al (2006) data set. The genes from these genomes were retrieved and aligned to the iTOL genes as a profile alignment in ClustalW v1.83 [30]. Phylogenetic hypotheses based on this alignment were then generated using Multiphyl [23] using the homogeneous (unpartitioned) model selection, tree reconstruction and bootstrap resampling capabilities of MultiPhyl.

### Ribosomal RNA Tree

16S rRNA sequences were obtained from the Ribosomal Database Project (RDP, [40]) or, when particular sequences were not available in the RDP, they were retrieved from GenBank (see table S1). All downloaded 16S rRNA genes were compared to the corresponding genes in our downloaded genomes to ensure the correct genes had been retrieved. The RDP alignment was used as a profile to align GenBank sequences using ClustalW v1.83 [30]. According to the AIC implemented in Modeltest [24], the best-fitting model of nucleotide substitution was the General Time Reversible (GTR) substitution model, with rates at variable sites sampled from a gamma distribution. Phylogeny reconstruction was carried out using the default TBR heuristic search in PAUP 4b10 [41]. Bootstrap resampling was also carried out using PAUP 4b10.

### Comparing trees and matrices

Pairwise Robinson-Foulds (RF) distances (symmetric-difference distances) [42] between trees were calculated using PAUP 4b10 [41]. Phylogenetic trees were visualized using TreeView [43] and TreeMap 2.0β [44] (see Additional file 1). Comparisons between distance matrices produced using the gene-content methods were performed by calculating a sum-of-squares distance. The matrices were transformed so that undefined values from the CR procedure were replaced with the largest value in the matrix. The resulting distance matrices could then be visualised using Principle Components Analysis (PCA) in the R statistical programming language (R Development Core Team, 2006).

## Results & discussion

Our objective was to explore a variety of exemplar analysis methods from each of the different categories of analysis type in order to ascertain whether variation in the resulting trees is trivial or extensive, random or accompanied by systematic bias. In the first instance, we used exploratory statistics to examine variation in the distance matrices produced by those methods that produced distance matrices. We focused on the CR approach and specifically, the effects that are seen with variation in the choice of conditioning genome.

### Variation within Conditioned Reconstruction approaches

**Figure 1:**
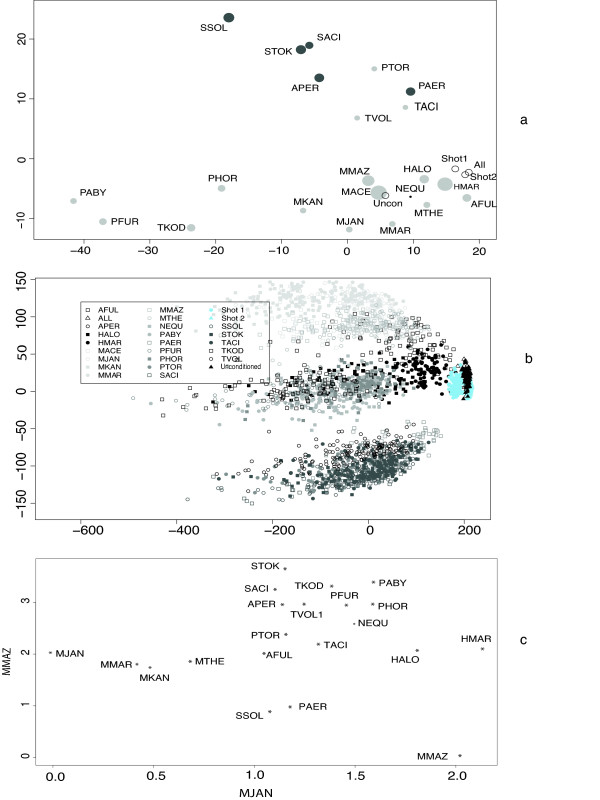
**1a displays a PCA of the variation between the distance matrices.** Each point is named after either the phylogenetic method used to create the matrix or in the case of CR the conditioning genome used. The size of the points is proportional to the size of the conditioning genome used. Figure 1b depicts PCA analysis of the 100 bootstrap replicates from each of the gene-content methods and the 22 variants of CR using one genome. Finally 1c displays a matrix produced using *Methanosarcina acetivorans *as the conditioning genome. The columns representing *Methanococcus jannaschii *and *Methanosarcina mazei *in the distance matrixhave been plotted. The dots on the plot represent the distance between the labeled genome and *Mco. jannaschii (*plotted on the x axis) and *Methanosarcina mazei (*plotted on the y-axis).

In the analysis of the CR approach it became obvious that the distance matrix that was recovered was very dependent on the conditioning genome that was used in the analysis. We inferred CR distance matrices using all possible (a total of 22) combinations of conditioning genomes. Another distance matrix was derived by taking a pair of genomes and calculating the distance between them using every other genome as a conditioning genome and then averaging these distances (Avg CR). Another distance matrix was produced using a synthetic conditioning genome where every gene family was present (unconditioned approach). Two final matrices were produced using the SHOT formulae in equations 1 and 2 above. We used PCA in order to visualize the most important sources of variation across the CR distance matrices as well as the two matrices from the SHOT methods. Figure 1a shows the most important axes following PCA of the distance matrices. Each point on the diagram represents the location of a distance matrix, with the relative closeness of points to one another being indicative of the relative similarities of the distance matrices. Beside each point is the name of the conditioning genome that was used in order to produce the matrix. In the case of the SHOT methods or the various CR alternatives, these are labeled appropriately. In this plot the points are proportional in size to the size of the conditioning genome and the colours of the shaded points are darker for Crenarchaeotes and lighter for Euryarchaeotes.

The two axes in this plot account for 74% of the total amount of variation in the PCA. The first axis (the abscissa) accounts for 52% of the variation and the second axis (the ordinate) accounts for 22% of the total variation. No other axis accounted for more than 6% of the variation, therefore these two axes are by far the most important correlates with variation in the distance matrices.

Firstly, an analysis of this plot shows that the same distance matrix is not produced every time and that the calculated distances heavily depend on the conditioning genome or the data treatment that is used. The most important trend in these data matrices (axis 1) is correlated with choice of conditioning genome. When conditioning genomes are used that are closely related, then the resulting distance matrices will tend to be closely related. For example, the distance matrices produced using the four Thermococci (see table S3 for classification) as the conditioning genomes are clustered together. A similar within-group clustering is observed when, say, the Thermoplasmatales, the Crenarchaeota or the Methanogens act as the conditioning genome. When *Archaeoglobus fulgidus *was used as the conditioning genome the resulting distance matrix also clustered with the Methanogens. *Arc. fulgidus *is a sulphur metabolising archaeon with similar biochemistry to the methanogens [45] so therefore, this placement is also perhaps not surprising. Therefore, taking an overall look at the results of using different conditioning genomes, we can see that phylogenetic position is the most important factor in inducing differences in the distance matrices.

The outliers on axis 1 in this plot are the matrices where the four Thermococci were used as conditioning genomes. These outliers account for much of the variation in axis 1. There are two things that can be said about these distance matrices. Firstly, three of these four matrices contained the highest proportion of undefined values in our analyses. Undefined values occur when attempting to perform an operation on invalid operands, *e.g. *getting the logarithm of a negative number. When one Thermococcus is chosen as the conditioning genome the distances between the other Thermococci and the rest of the genomes contain a high proportion of undefined values. This point is backed up by the claim [36] that a conditioning genome far from the taxon of interest is optimal. This may be the reason that these are outliers. Another possible reason is that these are close relatives so perhaps they are outliers because quite simply these four conditioning genomes have produced matrices that are similar to one another but very different to the rest of the conditioning genomes and the fact that they have large numbers of undefined values is purely incidental.

The second most important axis (axis 2) clearly defines the split between the Crenarchaeota and Euryarchaeota. So, even the second most important source of variation in the data is also related to phylogenetic affiliations of the conditioning genomes.

In order to explore whether these matrices are significantly different to one another we used bootstrap resampling of the data. For each dataset, we produced 100 bootstrap samples and 100 corresponding distance matrices. We expected to find one of two situations. Either the variation within 100 bootstrap replicates is so great that there is no correlation between matrices produced using a specific conditioning genome, or that the bootstrap replicates for a particular method would cluster together.

From figure 1b we can see that bootstrap samples tend to result in distance matrices that are generally similar to the matrices produced from the original data. Three clusters are observed in the bootstrapped data, one grouping comprises the Crenarchaeota, another consists of the Methanogens and the third contains the Thermococci. This means that the distance matrices derived from different treatments of the data are generally different but are clustered based on phylogenetic position. We interpret this to be significant support for a systematic bias as previously observed in two other studies [22,36].

Next, we analysed the phylogenetic trees that can be constructed from each of the different approaches. In this section, we present an exemplar analysis of what we see when we use one conditioning genome, when we combine information from different distance matrices to build a supertree [36] and also we present the results of comparisons of the different phylogenetic methods.

### Effect the Conditioning Genome has on its Closest Relatives

One recurring feature of phylograms that are constructed from matrices produced using the CR approach is the presence of a few long branches in the analysis (see supplementary material). A closer inspection of this phenomenon showed that the tree tips with unusual branch lengths corresponded usually with those taxa that are known to be close relatives of the conditioning genome. This is a systematic bias in CR [22,36]. When a phylogram was constructed using *Pyrococcus abyssi *as the conditioning genome (supplementary information), the three remaining Thermococci had elongated branch lengths in particular *P. horikoshii*. *P. horikoshii *is considered to be the closest relative to *P. abyssi *in the taxon sampling we have used. It has been reported that the two genomes share 1,122 kb in common and a similar chromosomal organisation, with an average amino acid identity of 77% [46]. The branch leading to *P. horikoshii *was 1.17 whilst the average branch length across the tree was 0.33 (see supplementary information, table S4 for branch lengths using other conditioning genomes).

Figure 1c is a plot of the distances from a matrix produced using *Methanosarcina acetivorans *as the conditioning genome. The dots on the plot represent the distance between the labeled genome and *Methanocococcus jannaschii *(plotted on the x-axis) and *Methanosarcina mazei *(plotted on the y-axis). Considering that both these genomes are methanogens, it would be expected that as the distance from a particular genome to *Mco. jannaschii *increases, an increase would also be observed in the distance from that genome to *Msa. mazei*. This scenario is evident in the supplementary material where *Mco. jannaschii *(x-axis) is plotted against *Methanococcus maripaludis *(y-axis). However there appears to be no such correlation in figure 1c. What would also be expected is that well recognized phylogenetic groups would cluster together on this plot. However this is also not observed, for example the Crenarchaeota do not form a cluster. The reason why this plot behaves differently is that the genome that has been plotted on the y-axis is *Msa. mazei*. *Msa. mazei *is the closest relative to the conditioning genome (*Msa. acetivorans*) in this analysis. It was inferred from a phylogram of this tree that the terminal branch leading to *Msa. mazei *had a length of 2.02 whilst the average branch length across the tree was only 0.31. It is also interesting to note that *M. mazei *features as an outlier on this plot, not with the other methanogens and it also has the second largest distance to *Mco. jannaschii*.

### Empirical example of choosing one Conditioning Genome

**Figure 2:**
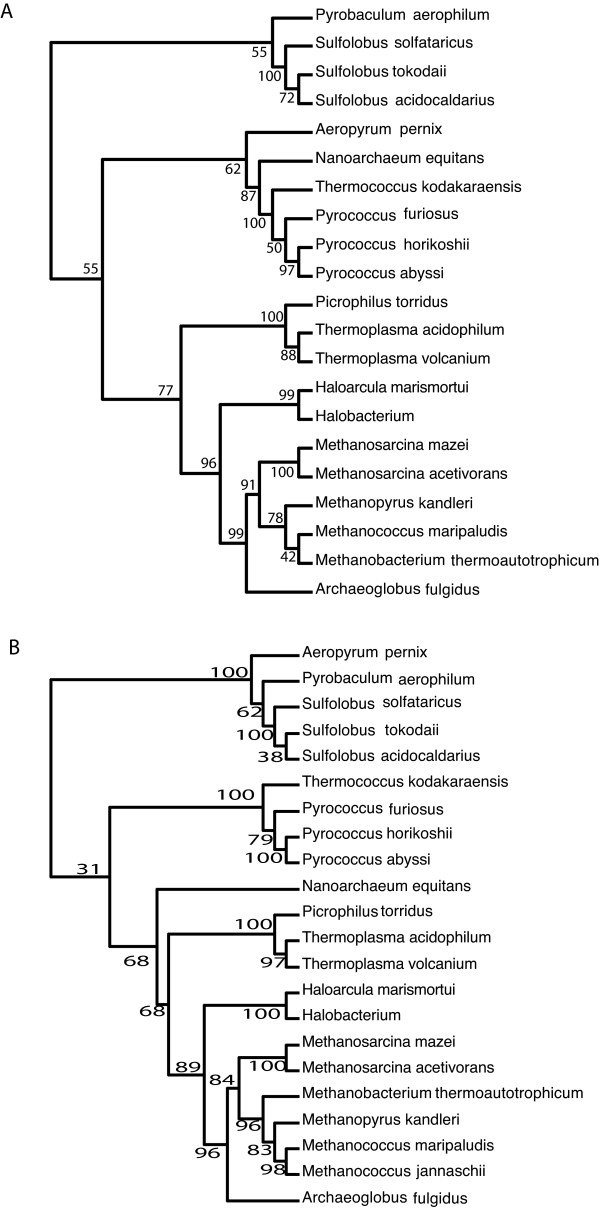
**(a) Phylogenetic tree constructed using the CR algorithm with only one conditioning genome chosen, in this case *Mco. jannaschii*.** The tree is rooted on four members of the Crenarchaeota (b) Phylogenetic tree constructed using Avg CR *i.e. *every genome in the analysis acts as the conditioning genome.

Figure 2a depicts a phylogenetic tree constructed using the neighbor-joining algorithm [47] with *Mco. jannaschii *chosen as the conditioning genome. The inferred neighbor-joining tree did not recover the Crenarchaeota as a clade (see table S3 for members of the Crenarchaeota). Four of the Crenarchaeota are found in a clade, however instead of featuring along with the other Crenarchaeota, *Aeropyrum pernix *appears in a group with the Themococci and *Nanoarchaeum equitans *(with bootstrap support of 62%), (see supplementary information for more examples of this scenario). It is interesting to note from figure 1a that *Mco. jannaschii *is the most outlying point on one end of axis 2.

### Comparing the Supertree (constructed using the modified BIONJ algorithm) to the Avg Conditioned Reconstruction tree

The trees derived using the modified BIONJ algorithm [36] were compared to the Avg CR tree and it was evident that they were quite similar. Both the inverse-variance weighting and the vote-counting tree differed from the CR tree by an RF distance of 6 [42]. The BIONJ supertree algorithm outperforms the Avg CR method as it has been stated the latter may not be tree-additive [36]. However the principles upon which the two methods are based are not entirely different; the supertree approach amalgamates information from the distance matrices produced using each of the 22 Archaea as the conditioning genome. In an approach that is not dissimilar, Avg CR produces only one distance matrix with its values being representative of the distances between two given taxa using the other twenty genomes as the conditioning genome, averaged by the number of genomes.

### Comparing different genome-phylogeny methods

**Figure 3:**
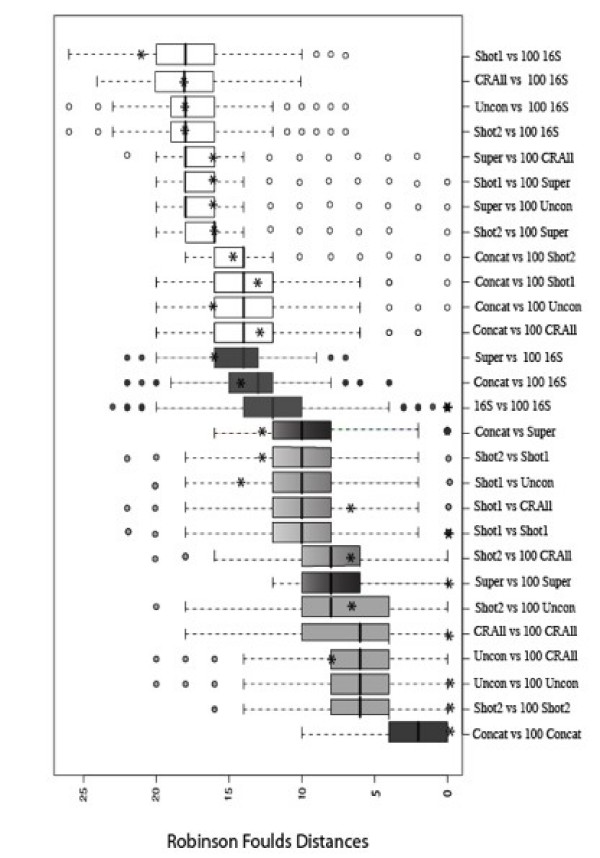
**Robinson Foulds distances between each of the 100 bootstrap replicates for a method against the sets of 100 bootstrap replicates for all the methods.** The sequenced-based versus sequenced-based methods are coloured black, the gene-content versus gene-content methods grey and the gene-content versus sequence-based are left blank. Each asterisk represents the Robinson Foulds distance between the unpermuted datasets.

In order to explore whether similar kinds of approaches such as gene-content methods or sequence-based methods gave similar or dissimilar results we undertook an analysis of RF [42] distances between trees produced by all the different methods (figure 3). Our specific comparison involved taking each of the 100 bootstrap replicates produced by a particular method and comparing every tree replicate with each of the sets of 100 bootstrap trees that had been constructed for all of the methods. For each method comparison (*i.e. *16S rRNA Vs Shot1) 10,000 individual comparisons were performed (100 × 100), resulting in 280,000 comparisons being performed in total across all the methods. Also plotted in this figure, represented by an asterisk is the RF [42] distance derived between the individual original trees derived using each of the methods. CR trees produced using only one conditioning genome were excluded from this analysis because these trees contain only 21 taxa with the conditioning genome being excluded from the phylogenetic analysis. Also excluded were the supertrees created using the modified BIONJ approach [36] as these were only included in the study to perform a comparison with the Avg CR tree. The results are presented in figure 3, where, for clarity, we have coloured the sequence-based versus sequence-based comparisons black, the gene-content versus gene-content analyses grey and the sequence-based versus gene-content comparisons are left open.

One single overriding trend emerges from this analysis. When we compare like with like (say, comparing gene-content with gene-content) the trees are more similar than when we compare trees derived using methods from different categories of phylogenetic approach (say, when comparing gene-content with sequence-based methods).

The extreme outliers are the comparison of each of the bootstrap replicates from the concatenated dataset against the same set of 100 bootstrap replicates. The extreme outlier on the other side is found in the comparison of the 100 16S rRNA bootstrapped gene trees with 100 bootstrap replicates created using the SHOT 1 method.

Figure 3 illustrates that the average number of steps (e.g. merging or splitting nodes) involved in transforming the bootstrapped 16S rRNA tree replicates into the concatenated alignment replicates is 13 (*i.e. *the RF distance). To transform the 16S rRNA tree replicates into the supertree replicates requires an average of 14 elementary operations and to convert the 16S rRNA replicates into the SHOT variant 1 replicates is 18. Clearly these methods produce significantly different tree topologies.

A visual comparison of the 16S rRNA versus the Conditioned Reconstruction tree is provided using the TreeMap 2.0β software [44] in the supplementary material (TreeMap folder). Perhaps at a first glance the trees do not seem significantly different but if examined in greater detail using either distance metrics or indeed by eye it is evident that fundamental differences arise. Take for example the topology of the Thermococci, the placement of *Nanoarchaeum equitans *or the position of *Methanopyrus kandleri *to name but a few.

### What the trees say

Having different principles upon which the methods are based is inevitably going to lead to differences, for example within the Archaeal phylogeny a methanogen that appears in conflicting positions in the trees created using the different methods is *Methanopyrus kandleri*. It is known that whole-genome trees based on either gene-content or on the conservation of gene order commonly group *M. kandleri *with the Methanococcales and the Methanobacteriales, because *M. kandleri *is known to share groups of genes associated with methanogenesis and to have a similar operon organisation to *Mco. jannaschii *and *M. thermoautotrophicum *[48]. It is therefore not surprising that in each of the four whole-genome trees such a positioning was observed and backed up with high bootstrap support.

Methods that are not based on gene-content do not place such a strong emphasis on shared genes such as those involved in methanogenesis, perhaps for this reason instead of grouping with the other methanogens in the supertree *M. kandleri *is found in a clade with *Arc. fulgidus*.

## Conclusion

Conclusions about the similarities of phylogenetic trees is often carried out using visual assessment, without using any explicit measure of tree-to-tree similarity. As an example, Choi and Kim [49] claimed that phylogenetic trees reconstructed by most methods are "practically the same as that constructed from SSU rRNA sequences". In this study, we were not trying to evaluate the method of phylogeny reconstruction or to infer the "Tree of Life", if one exists [50]. Rather, we wished to explore variation in the resulting tree topology when a number of exemplar phylogenetic approaches were examined.

Even though the authors of CR claim their method is robust to HGT, the Avg CR method performs very similarly to the other gene-content methods. We have found that CR using one conditioning genome is misled by genome size and the taxonomy of the conditioning genome. This can have a profound affect on a number of aspects of the analysis. First of all, the choice of conditioning genome affects the inferred distances between two other genomes. Depending on which conditioning genome is used, two organisms can appear to be close relatives or distant relatives. In addition, there is a systematic bias in the inferred distance between two genomes if these two genomes are close relatives of the conditioning genome.

The case of the effect of the conditioning genome on the inferred distance can be shown in the following hypothetical situation. Consider two similar genomes of interest, say, two species of the same genus. They have very similar genome content. Now consider two conditioning genomes, one of whom is also in the same genus and one that is in a different phylum. In both cases, the conditioning genome has 100 genes. The presence-absence matrix of genes in the genomes of interest that is recorded when using the conditioning genome that is in the same genus is as follows (left-to-right: presence, absence; top-to-bottom: presence, absence):

$$\left[\begin{array}{cc} 96 & 2\\ 0 & 2 \end{array}\right]$$

The matrix for the same two genomes when using a conditioning genome that is a distant relative could be as follows:

$$\left[\begin{array}{cc} 49 & 1\\ 1 & 49 \end{array}\right]$$

The LogDet distance estimated from the first matrix is 0.357 whereas the distance estimated from the second matrix is 0.041. This explains why we see phylogenetic trees with long branches leading to those taxa that are close relatives of the conditioning genome. This is a systematic error in the CR method [22,36]. This kind of error has been noted previously and there have been suggestions for how to avoid this error [36]. However, given the pervasiveness of the problem, it is not obvious to us that simply being judicious about the choice of conditioning genome will solve the problem.

Of concern also is the comparison of the 100 bootstrapped phylogenetic trees derived from the concatenated data against the same set of tree 100 trees. This is the single longest alignment in our analyses and it is also the dataset where there was the closest resemblance between the bootstrap samples. The real meaning of bootstrapping has been debated, but probably the most accurate assessment of what it does is that it evaluates whether there has been enough sampling of the original data [9]. The data in figure 3 seem to indicate that in general during bootstrapping a similar phylogenetic tree was returned. Clearly, however, from the other analyses, there are conflicts in the genome data that are not adequately accounted for by the concatenated data. We would go further to suggest that the concatenated alignment is in effect hiding the variation in the genome-scale data. Indeed, it has been suggested that phylogenetic trees derived using this approach represent the "tree of one percent" of a genome [51]. It might not be surprising therefore that there is considerable agreement among bootstrap samples from a large number of alignment columns that have been pre-selected from a specific group of informational genes. This does not mean that we can infer from such a dataset that there is a robust phylogeny. We can infer that using a dataset of informational genes, where obvious horizontal transfers have been removed, bootstrapping really is a measure of whether we will benefit from sampling more alignment columns and using them in the same kind of analysis.

The main point raised by our analysis is that while different clustering methods have been developed that use different data, these methods produce different inferences and these inferences are not essentially the same as the inferences from SSU rRNA (see [49], where the opposite claim is made). The gene-content methods produce results that are similar to one another. We suggest that these phylogenies are biased towards metabolism-based classifications (i.e. the clustering of organisms based on similarity of metabolism) rather than on the basis of recentness of common ancestry, although definite elements of both come through in the same analysis. Phylogenetic trees derived from the SSU rRNA gene or from the concatenated alignment are really trees of small amounts of any genome and these alignment positions are likely to be in good agreement with one another, being generally inherited together. The evolutionary history of the remainder of the genomes is however neglected. The supertree approach seeks to amalgamate the information from many input trees and therefore is a sequence-based method that is more democratic in terms of getting its information. However, the data analysed here represent a small fraction of the total amount of the evolutionary information that is available and until liberal supertree methods can accommodate complete genome information, they too, will only infer histories from partial data. In short, none of the phylogenetic trees presented here are without merit, however, interpretation of these trees should always be accompanied by caveats that describe specifically what the trees represent. It is still not clear that in any of these instances the end product is a true species phylogeny. What is clear is that these methods do not agree with one another. We feel it is unwise to speculate on which method, model, approach or encoding of a genome is likely to be the best – this may be a task for tree interpretation in any case – however in the future it is possible that appropriate simulation studies will provide insights into what each method really recovers.

## Authors' contributions

AMc performed all the analysis of the methods and wrote the computer code required drafted the manuscript. JC performed the PCA analysis and provided help with the R software package and editing the manuscript. JMc conceived the idea for this project, oversaw this study and in particular edited the manuscript. All authors have read and approved the final manuscript.

## Supplementary Material

Additional file 1 Supplementary materials. Click here for file
