# Supplementary material for: The tree of genomes: An empirical comparison of genome-phylogeny reconstruction methods
Source: BMC Evol Biol. 2008 Nov 12;8:312. doi: 10.1186/1471-2148-8-312 (PMC2592249; doi:10.1186/1471-2148-8-312)
Supplement: Additional file 1 — Supplementary materials. [file 1471-2148-8-312-S1.gz › SupplementaryMaterial/Phylogram and Plotted Distance Matrix.tar!/Phylogram and Plotted Distance Matrix/MJANvsMMARDistanceMatrix.doc]

**Figure 2:** Distance matrix with column MJAN (*Methanococcus jannaschii)* plotted against column MMAR (*Methanococcus maripaludis)*. The above plot shows the distance of labeled genomes to MJAN on the x-axis, and MMAR on the y-axis. There is an obvious correlation; when the distance from a particular genome to MJAN increases an increase is also observed to MMAR, as would be expected amongst members of the Methanogens. Also evident is that related genomes had similar distances to the two genomes under analysis here, for example the Crenarchaeota are all located clustered in the middle of the plot.
