# Supplementary material for: The tree of genomes: An empirical comparison of genome-phylogeny reconstruction methods
Source: BMC Evol Biol. 2008 Nov 12;8:312. doi: 10.1186/1471-2148-8-312 (PMC2592249; doi:10.1186/1471-2148-8-312)
Supplement: Additional file 1 — Supplementary materials. [file 1471-2148-8-312-S1.gz › SupplementaryMaterial/Phylogram and Plotted Distance Matrix.tar!/Phylogram and Plotted Distance Matrix/Phylogram.doc]

**Figure 1. A phylogram using *P. abyssi* as the conditioning genome**
