# Supplementary material for: The tree of genomes: An empirical comparison of genome-phylogeny reconstruction methods
Source: BMC Evol Biol. 2008 Nov 12;8:312. doi: 10.1186/1471-2148-8-312 (PMC2592249; doi:10.1186/1471-2148-8-312)
Supplement: Additional file 1 — Supplementary materials. [file 1471-2148-8-312-S1.gz › SupplementaryMaterial/README For Supp.doc]

Readme for supplementary material of paper entitled "The Tree of Genomes: An Empirical Comparison of Genome-Phylogeny Reconstruction Methods".

***Folder 1=AlignmentToMake16SrRNAtree.***

This folder contains an alignment of the twenty-two 16S rRNA sequences that were either downloaded from the Ribosomal Database Project or Genbank; the file is in nexus format.

***Folder 2=ConcatenatedAlignment.***

This folder contains the concatenated alignment of 31 genes. The file is in fasta format.

***Folder 3=DifferentConditioningGenomes.***

This folder contains the 22 different trees constructed when different conditioning genomes were chosen; each file is named after the genome that was selected as the conditioning genome. The trees are in newick format.

***Folder 4=InputTreesForSuperTree.***

This folder contains the individual trees that were amalgamated to form the Supertree, a tree exists for every single gene family used.

***Folder 5=Table.***

This folder contains four tables:

*Table 1* relates to the downloading of the 16S rRNA genes for each genome (i.e. whether it was downloaded from the Ribosomal Database Project or if it had to be obtained from GenBank).

*Table 2* details the 31 genes used to construct the concatenated alignment.

*Table 3* contains the classification of the 22 Archaea into groups.

*Table 4* contains information about the elongated branch lengths amongst the closest relatives of the chosen conditioning genome.

***Folder 6=TreeMap***

This folder contains six tree comparisons displayed in TreeMap 2.0β (Page & Charleston, 2002). The trees provide visual comparisons between the Avg Conditioned Reconstruction tree and the six other phylogenetic methods used in the analysis.

***Folder 7= TreeProducedFromBionjAlgorithm***

This folder contains the two trees produced using the modified BIONJ approach; one tree is produced using inverse-variance weighting and the other using vote-counting.

***Folder 8=Phylogram and Plotted Distance Matrix***

This folder contains a phylogram constructed using *Pyrococcus abysii* as the conditioning genome; elongated branch lengths are evident in the branches leading to the remaining Thermococci.

Also contained within this folder is a distance matrix derived using *Methanosarcina acetivorans* as the conditioning genome. The columns representing *Methanococcus jannaschii and Methanococcus maripaludis* are plotted.
