# Supplementary material for: The tree of genomes: An empirical comparison of genome-phylogeny reconstruction methods
Source: BMC Evol Biol. 2008 Nov 12;8:312. doi: 10.1186/1471-2148-8-312 (PMC2592249; doi:10.1186/1471-2148-8-312)
Supplement: Additional file 1 — Supplementary materials. [file 1471-2148-8-312-S1.gz › SupplementaryMaterial/Table/Table S1.doc]

***Table S1***.

| ***Cogent code*** | **Species name** | **RDP** | **Genbank Acc. No**. |
| --- | --- | --- | --- |
| *MJAN-DSM-01* | *Methanococcus jannaschii DSM2661* | P | - |
| *MTHE-DEL-01* | *Methanobacterium thermoautotrophicum* | P | - |
| *AFUL-DSM-01* | *Archaeoglobus fulgidus DSM4304* | P | - |
| *PHOR-OT3-01* | *Pyrococcus horikoshii OT3* | P | - |
| *APER-XK1-01* | *Aeropyrum pernix K1* | P | - |
| *SSOL-XP2-01* | *Sulfolobus solfataricus P2* | P | - |
| *PAER-IM2-01* | *Pyrobaculum aerophilum IM2* | P | - |
| *MKAN-AV1-01* | *Methanopyrus kandleri AV19* | P | - |
| *MACE-C2A-01* | *Methanosarcina acetivorans C2A* | P | - |
| *PFUR-638-01* | *Pyrococcus furiosus DSM3638* | P | - |
| *MMAZ-GO1-01* | *Methanosarcina mazei Go1* | P | - |
| *SACI-639-01* | *Sulfolobus acidocaldarius DSM 639* | P | - |
| *TVOL-GSS-01* | *Thermoplasma volcanium GSS1* | A | AJ299215 |
| *PABY-GE5-01* | *Pyrococcus abyssi GE5* | A | Z70246 |
| *TACI-DSM-01* | *Thermoplasma acidophilum DSM1728* | A | NC_002578 |
| *HALO-NRC-01* | *Halobacterium sp. NRC-1* | A | NC_002607 |
| *STOK-XX7-01* | *Sulfolobus tokodaii str. 7* | A | BA000023 |
| *NEQU-N4M-01* | *Nanoarchaeum equitans Kin4-M* | A | AJ318041 |
| *PTOR-790-01* | *Picrophilus torridus DSM9790* | A | NC_005877 |
| *MMAR-XS2-01* | *Methanococcus maripaludis S2* | A | NC_005791 |
| *HMAR-502-01* | *Haloarcula marismortui ATCC43049* | A | AF034620 |
| *TKOD-OD1-01* | *Thermococcus kodakaraensis KOD1* | A | NC_006624 |

**Legend:**

Information concerning the source of the 16S rRNA gene for the 22 Archaeal genomes. P indicates (present) i.e. the gene was available from the Ribosomal Database Project for this strain, and an A (absent) the opposite. In cases where the 16S rRNA was downloaded from GenBank accession numbers are given.
