# Supplementary material for: The tree of genomes: An empirical comparison of genome-phylogeny reconstruction methods
Source: BMC Evol Biol. 2008 Nov 12;8:312. doi: 10.1186/1471-2148-8-312 (PMC2592249; doi:10.1186/1471-2148-8-312)
Supplement: Additional file 1 — Supplementary materials. [file 1471-2148-8-312-S1.gz › SupplementaryMaterial/Table/Table S2.doc]

**Table S2.** Universally distributed COGs used in this study (Ciccarelli *et al.,* 2006)

| **Orthologous Goup** | **Annotation** |
| --- | --- |
| COG0012 | Predicted GTPase, probable translation factor |
| COG0016 | Phenylalanine-tRNA synthethase alpha subunit |
| COG0048 | Ribosomal protein S12 |
| COG0049 | Ribosomal protein S7 |
| COG0052 | Ribosomal protein S2 |
| COG0080 | Ribosomal protein L11 |
| COG0081 | Ribosomal protein L1 |
| COG0087 | Ribosomal protein L3 |
| COG0091 | Ribosomal protein L22 |
| COG0092 | Ribosomal protein S3 |
| COG0093 | Ribosomal protein L14 |
| COG0094 | Ribosomal protein L5 |
| COG0096 | Ribosomal protein S8 |
| COG0097 | Ribosomal protein L6P/L9E |
| COG0098 | Ribosomal protein S5 |
| COG0099 | Ribosomal protein S13 |
| COG0100 | Ribosomal protein S11 |
| COG0102 | Ribosomal protein L13 |
| COG0103 | Ribosomal protein S9 |
| COG0172 | Seryl-tRNA synthetase |
| COG0184 | Ribosomal protein S15P/S13E |
| COG0186 | Ribosomal protein S17 |
| COG0197 | Ribosomal protein L16/L10E |
| COG0200 | Ribosomal protein L15 |
| COG0201 | Preprotein translocase subunit SecY |
| COG0202 | DNA-directed RNA polymerase, alpha subunit |
| COG0256 | Ribosomal protein L18 |
| COG0495 | Leucyl-tRNA synthetase |
| COG0522 | Ribosomal protein S4 and related proteins |
| COG0525 | Valyl-tRNA synthetase |
| COG0533 | Metal-dependent proteases with chaperone activity |
