# Supplementary material for: The tree of genomes: An empirical comparison of genome-phylogeny reconstruction methods
Source: BMC Evol Biol. 2008 Nov 12;8:312. doi: 10.1186/1471-2148-8-312 (PMC2592249; doi:10.1186/1471-2148-8-312)
Supplement: Additional file 1 — Supplementary materials. [file 1471-2148-8-312-S1.gz › SupplementaryMaterial/Table/Table S3.doc]

***Table S3***.

| Species name | Classification |
| --- | --- |
| *Methanococcus jannaschii DSM2661* | Methanococcales |
| *Methanococcus maripaludis S2* | Methanococcales |
| *Methanobacterium thermoautotrophicum* | Methanobacteria |
| *Methanopyrus kandleri AV19* | Methanopyrales |
| *Methanosarcina acetivorans C2A* | Methanosarcinales |
| *Methanosarcina mazei Go1* | Methanosarcinales |
| *Pyrococcus horikoshii OT3* | Thermococcales |
| *Pyrococcus abyssi GE5* | Thermococcales |
| *Thermococcus kodakaraensis KOD1* | Thermococcales |
| *Pyrococcus furiosus DSM3638* | Thermococcales |
| *Aeropyrum pernix K1* | Desulfurococcales (Crenarcheota) |
| *Pyrobaculum aerophilum IM2* | Thermoproteaceae (Crenarcheota) |
| *Thermoplasma volcanium GSS1* | Thermoplasmata |
| *Picrophilus torridus DSM9790* | Thermoplasmata |
| *Thermoplasma acidophilum DSM1728* | Thermoplasmata |
| *Sulfolobus solfataricus P2* | Sulfolobales (Crenarcheota) |
| *Sulfolobus acidocaldarius DSM 639* | Sulfolobales (Crenarcheota) |
| *Sulfolobus tokodaii str. 7* | Sulfolobales (Crenarcheota) |
| *Halobacterium sp. NRC-1* | Halobacteria |
| *Haloarcula marismortui ATCC43049* | Halobacteria |
| *Archaeoglobus fulgidus DSM4304* | Archaeoglobales |
| *Nanoarchaeum equitans Kin4-M* | Nanoarchaeum |
