# Supplementary material for: The tree of genomes: An empirical comparison of genome-phylogeny reconstruction methods
Source: BMC Evol Biol. 2008 Nov 12;8:312. doi: 10.1186/1471-2148-8-312 (PMC2592249; doi:10.1186/1471-2148-8-312)
Supplement: Additional file 1 — Supplementary materials. [file 1471-2148-8-312-S1.gz › SupplementaryMaterial/Table/Table S4.doc]

| Conditioning Genome Name | Average Branch Length | Length to Closest Relative(s) |
| --- | --- | --- |
| *Methanococcus jannaschii DSM2661* | 0.26 | 0.55 |
| *Methanobacterium thermoautotrophicum* | 0.25 | 0.42 |
| *Archaeoglobus fulgidus DSM4304* | 0.27 | 0.70 |
| *Pyrococcus horikoshii OT3* | 0.29 | 0.40 |
| *Aeropyrum pernix K1* | 0.30 | 0.85 |
| *Sulfolobus solfataricus P2* | 0.32 | 0.97 |
| *Pyrobaculum aerophilum IM2* | 0.30 | 0.83 |
| *Methanopyrus kandleri AV19* | 0.26 | 0.81 |
| *Methanosarcina acetivorans C2A* | 0.31 | 2.03 |
| *Pyrococcus furiosus DSM3638* | 0.32 | .99 |
| *Methanosarcina mazei Go1* | 0.33 | 1.89 |
| *Sulfolobus acidocaldarius DSM 639* | 0.33 | 0.92 |
| *Thermoplasma volcanium GSS1* | 0.29 | 0.75 |
| *Pyrococcus abyssi GE5* | 0.33 | 1.17 |
| *Thermoplasma acidophilum DSM1728* | 0.29 | 0.61 |
| *Halobacterium sp. NRC-1* | 0.29 | 1.56 |
| *Sulfolobus tokodaii str.* | 0.31 | 0.65 |
| *Picrophilus torridus DSM9790* | 0.27 | 1.17 |
| *Methanococcus maripaludis S2* | 0.28 | 0.68 |
| *Haloarcula marismortui ATCC43049* | 0.26 | 0.96 |
| *Thermococcus kodakaraensis KOD1* | 0.32 | 1.5 |

**Table S4**

**Legend:**

The above table provides information about branch lengths. The left hand column represents the conditioning genome that was used in each analysis to build the tree. The middle column is the average branch length across that tree and the right column is the branch length linking the conditioning genome to its closest relative(s).
